# Severity and risk factors of interval breast cancer in Queensland, Australia: a population-based study

**DOI:** 10.1007/s12282-023-01439-4

**Published:** 2023-02-21

**Authors:** Kou Kou, Jessica Cameron, Philippa Youl, Chris Pyke, Suzanne Chambers, Jeff Dunn, Joanne F. Aitken, Peter D. Baade

**Affiliations:** 1 Cancer Council Queensland, Spring Hill, PO Box 201, Brisbane, QLD 4001 Australia; 2 School of Mathematical Sciences, Queensland University of Technology, Brisbane, Australia; 3 Cancer Alliance Queensland, Metro South Hospital and Health Service, Woolloongabba, Australia; 4 Mater Hospitals South Brisbane, Brisbane, Australia; 5 Faculty of Health, University of Technology Sydney, Sydney, Australia; 6 Prostate Cancer Foundation of Australia, Sydney, Australia; 7 School of Public Health, The University of Queensland, Brisbane, Australia; 8 School of Public Health and Social Work, Queensland University of Technology, Brisbane, Australia; 9 Institute for Resilient Regions, University of Southern Queensland, Brisbane, Australia; 10 Centre for Data Science, Faculty of Science, Queensland University of Technology, Brisbane, Australia; 11 Menzies Health Institute Queensland, Griffith University, Gold Coast Campus, Parklands Drive, Southport, QLD Australia

**Keywords:** Breast cancer, Interval cancer, Mammogram screening, Severity, Risk factors

## Abstract

**Background:**

Interval breast cancers (BC) are those diagnosed within 24 months of a negative mammogram. This study estimates the odds of being diagnosed with high-severity BC among screen-detected, interval, and other symptom-detected BC (no screening history within 2 years); and explores factors associated with being diagnosed with interval BC.

**Methods:**

Telephone interviews and self-administered questionnaires were conducted among women (*n* = 3,326) diagnosed with BC in 2010–2013 in Queensland. Respondents were categorised into screen-detected, interval, and other symptom-detected BCs. Data were analysed using logistic regressions with multiple imputation.

**Results:**

Compared with screen-detected BC, interval BC had higher odds of late-stage (OR = 3.50, 2.9–4.3), high-grade (OR = 2.36, 1.9–2.9) and triple-negative cancers (OR = 2.55, 1.9–3.5). Compared with other symptom-detected BC, interval BC had lower odds of late stage (OR = 0.75, 0.6–0.9), but higher odds of triple-negative cancers (OR = 1.68, 1.2–2.3). Among women who had a negative mammogram (*n* = 2,145), 69.8% were diagnosed at their next mammogram, while 30.2% were diagnosed with an interval cancer. Those with an interval cancer were more likely to have healthy weight (OR = 1.37, 1.1–1.7), received hormone replacement therapy (2–10 years: OR = 1.33, 1.0–1.7; > 10 years: OR = 1.55, 1.1–2.2), conducted monthly breast self-examinations (BSE) (OR = 1.66, 1.2–2.3) and had previous mammogram in a public facility (OR = 1.52, 1.2–2.0).

**Conclusion:**

These results highlight the benefits of screening even among those with an interval cancer. Women-conducted BSE were more likely to have interval BC which may reflect their increased ability to notice symptoms between screening intervals.

**Supplementary Information:**

The online version contains supplementary material available at 10.1007/s12282-023-01439-4.

## Introduction

Interval breast cancers in Australia are defined as a breast cancer diagnosed less than 24 months after a negative mammography screen [1]. Interval cancers are typically caused by rapid and aggressive tumour growth; however, some result from a false negative in the previous mammogram [2, 3].

Australian and international studies report that, compared to screen-detected breast cancers, interval breast cancers are characterised by more advanced stage at diagnosis, larger tumour size, higher grade, higher proportion of triple-negative tumours [3–9], and lower survival [10, 11]. However, it has not been previously reported whether the severity of interval breast cancers differ from those of breast cancers diagnosed by symptoms among women with no screening history within two years of diagnosis.

Factors shown to be associated with a higher risk of interval breast cancer include the use of hormone replacement therapy (HRT), family history of breast cancer, high breast density, not being overweight, and younger age [4, 5, 9, 12–17]. However, each of these studies included only a limited number of potential factors. In addition, although clinical and self-examination history, lifestyle, family history of breast cancer, reproductive history, and socioeconomic status (SES) factors play a role in the development of BC [18–21], their association with interval cancer have not been fully investigated.

Using data from a large cohort study of breast cancer patients diagnosed in Queensland, Australia during 2010–2013, this study aimed to explore whether interval breast cancers differed to other symptom-detected breast cancers and to screen-detected breast cancers with regard to severity at diagnosis; and explores factors associated with being diagnosed with interval BC when compared with screen-detected cancers.

## Patients and methods

### Study population

The Breast Cancer Outcomes Study (BCOS) [1, 22, 23] is a longitudinal study among women aged 20 to 79 years diagnosed with invasive breast cancer in Queensland, Australia between 1 March 2010 and 30 June 2013. Of the 5,426 eligible women identified for a telephone interview, 66 were deceased, treating doctors did not provide consent to approach 688 women and 3,326 (71.2% having doctor consent) completed the interview. The telephone interview was completed less than 3 years after diagnosis, with more than half completing the interview within 391 days [23]. There was no evidence of a difference in age distribution between participants and non-participants; however, women diagnosed with advanced disease or living in major cities were less likely to participate [22]. A study published by the authors in 2020 used data from the same cohort [23].

All participating women were categorised into one of three groups according to how their breast cancer was diagnosed. Screen-detected patients were those where the suspicion of breast cancer was made via routine mammography or ultrasound screening examination [22]. Symptom-detected patients were those whose first sign or symptom of breast cancer was initially noticed by themselves, a doctor, or a layperson. Symptom-detected patients were further divided into “interval breast cancer patients” (whose interval between date of last negative mammogram and date of diagnosis was 2 years, i.e. 730 days, or less) and “other symptom-detected patients” (who have no history of a mammogram or whose interval between date of last negative mammogram and date of diagnosis was more than 2 years).

### Individual-level variables

Individual-level variables included clinical factors such as age at diagnosis, cancer stage, grade, oestrogen receptor (ER) status, progesterone receptor (PR) status, and human epidermal growth factor receptor 2 (HER2) status. The clinical severity indicator, ‘triple negative (ER-, PR-, HER2-)’, was generated based on the status of the three receptors (Table 1) [24]. In addition, factors regarding clinical breast and self-examination history, lifestyle factors, reproductive history, family history of breast and ovarian cancer, and SES were also included in the analysis.

**Table 1:** Number (column %) of screen-detected, interval, and other symptom-detected breast cancer patients by different factors

	Overall	Screen-detected^1^	Interval cancer^2^	Other symptom-detected^3^	*p* value^4^
**All**	**3326 (100)**	**1642 (49.4)**	**660 (19.8)**	**1024 (30.8)**	
Age of diagnosis					
< 50	897 (27.0)	239 (14.6)	167 (25.3)	491 (48.0)	< 0.01
50–59	946 (28.4)	498 (30.3)	222 (33.6)	226 (22.1)	
60–69	1026 (30.9)	652 (39.7)	191 (28.9)	183 (17.9)	
70–79	457 (13.7)	253 (15.4)	80 (12.1)	124 (12.1)	
Clinical severity indicators					
Stage					
Stage I	1601 (48.1)	1083 (66.0)	231 (35.0)	287 (28.0)	< 0.01
Stage II–IV	1666 (50.1)	552 (33.6)	412 (62.4)	702 (68.6)	
Missing	59 (1.8)	7 (0.4)	17 (2.6)	35 (3.4)	
Grade					
Grade 1–2	2205 (66.3)	1267 (77.2)	377 (57.1)	561 (54.8)	< 0.01
Grade 3	1076 (32.4)	368 (22.4)	271 (41.1)	437 (42.7)	
Missing	45 (1.4)	7 (0.4)	12 (1.8)	26 (2.5)	
Triple negative					
No	2917 (87.7)	1504 (91.6)	530 (80.3)	883 (86.2)	< 0.01
Yes	263 (7.9)	90 (5.5)	86 (13.0)	87 (8.5)	
Missing	146 (4.4)	48 (2.9)	44 (6.7)	54 (5.3)	
Clinical and self-examination history					
Clinical breast examination (CBE)					
At least annually	808 (24.3)	455 (27.7)	192 (29.1)	161 (15.7)	< 0.01
Irregularly	1877 (56.4)	921 (56.1)	364 (55.2)	592 (57.8)	
Never	612 (18.4)	266 (16.2)	97 (14.7)	249 (24.3)	
Missing	29 (0.9)	0 (0)	7 (1.1)	22 (2.2)	
Breast self-examination (BSE)					
At least monthly	928 (27.9)	463 (28.2)	207 (31.4)	258 (25.2)	<0.01
Irregularly	1881 (56.6)	905 (55.1)	382 (57.9)	594 (58.0)	
Never	508 (15.3)	274 (16.7)	69 (10.5)	165 (16.1)	
Missing	9 (0.3)	0 (0)	2 (0.3)	7 (0.7)	
Lifestyle					
BMI^5^					
Healthy weight	1292 (38.9)	547 (33.3)	288 (43.6)	457 (44.6)	< 0.01
Overweight	2034 (61.2)	1095 (66.7)	372 (56.4)	567 (55.4)	
Physical activity					
Insufficient	1444 (43.4)	724 (44.1)	257 (38.9)	463 (45.2)	< 0.01
Sufficient	1835 (55.2)	898 (54.7)	402 (60.9)	535 (52.3)	
Missing	47 (1.4)	20 (1.2)	1 (0.2)	26 (2.5)	
Smoking					
Current smoker	266 (8.0)	109 (6.6)	37 (5.6)	120 (11.7)	< 0.01
Used to smoke	1169 (35.2)	575 (35.0)	241 (36.5)	353 (34.5)	
Never smoked	1886 (56.7)	958 (58.3)	381 (57.7)	547 (53.4)	
Missing	5 (0.2)	0 (0)	1 (0.2)	4 (0.4)	
Drinking					
< Once a month	1293 (38.9)	620 (37.8)	229 (34.7)	444 (43.4)	< 0.01
≥ Once a month	2033 (61.1)	1022 (62.2)	431 (65.3)	580 (56.6)	
Reproductive history					
Age at menarche					
≤ 13	2160 (64.9)	1058 (64.4)	424 (64.2)	678 (66.2)	0.15
> 13	1131 (34.0)	568 (34.6)	233 (35.3)	330 (32.2)	
Missing	35 (1.1)	16 (1.0)	3 (0.5)	16 (1.6)	
Duration of menstruation					
≤ 35 years	1639 (49.3)	690 (42.0)	325 (49.2)	624 (60.9)	< 0.01
> 35 years	1601 (48.1)	908 (55.3)	323 (48.9)	370 (36.1)	
Missing	86 (2.6)	44 (2.7)	12 (1.8)	30 (2.9)	
Menopause					
Yes	2691 (80.9)	1430 (87.1)	549 (83.2)	712 (69.5)	< 0.01
No	635 (19.1)	212 (12.9)	111 (16.8)	312 (30.5)	
Age started using contraceptives					
Never used	413 (12.4)	212 (12.9)	63 (9.6)	138 (13.5)	< 0.01
≤ 18	1062 (31.9)	400 (24.4)	221 (33.5)	441 (43.1)	
> 18	1835 (55.2)	1017 (61.9)	376 (57.0)	442 (43.2)	
Missing	16 (0.5)	13 (0.8)	0 (0)	3 (0.3)	
Duration of contraceptives using					
Never or < 2 years	688 (20.7)	355 (21.6)	119 (18.3)	214 (20.9)	0.25
2–10 years	1301 (39.1)	655 (39.9)	265 (40.2)	381 (37.2)	
> 10 years	1318 (39.6)	621 (37.8)	272 (41.2)	425 (41.5)	
Missing	19 (0.6)	11 (0.7)	4 (0.6)	4 (0.4)	
Age at first childbirth					
Never give birth	112 (3.4)	50 (3.1)	25 (3.8)	37 (3.6)	< 0.01
≤ 30	2358 (70.9)	1216 (74.1)	485 (73.5)	657 (64.2)	
> 30	467 (14.0)	199 (12.1)	92 (13.9)	176 (17.2)	
Missing	389 (11.7)	177 (10.8)	58 (8.8)	154 (15.0)	
Number of children					
None	112 (3.4)	50 (3.1)	25 (3.8)	37 (3.6)	0.62
1–2	1554 (46.7)	755 (46.0)	306 (46.4)	493 (48.1)	
> 2	1660 (49.9)	837 (51.0)	329 (49.9)	494 (48.2)	
Duration of breastfeeding					
None	957 (28.8)	460 (28.0)	175 (26.5)	322 (31.5)	0.04
1–12 months	1157 (34.8)	602 (36.7)	223 (33.8)	332 (32.4)	
> 12 month	1212 (36.4)	580 (35.3)	262 (39.7)	370 (36.1)	
Duration of hormone replacement therapy (HRT)					
Never or < 2 years	2441 (73.4)	1110 (67.6)	447 (67.7)	884 (86.3)	< 0.01
2–10 years	515 (15.5)	313 (19.1)	126 (19.1)	76 (7.4)	
> 10 years	290 (8.7)	167 (10.2)	75 (11.4)	48 (4.7)	
Missing	80 (2.4)	52 (3.2)	12 (1.8)	16 (1.6)	
Family history					
Relatives with breast or ovarian cancer					
None	1713 (51.5)	811 (49.4)	325 (49.2)	577 (56.4)	< 0.01
2nd degree	686 (20.6)	319 (19.4)	139 (21.1)	228 (22.3)	
1st degree	417 (12.5)	236 (14.4)	81 (12.3)	100 (9.8)	
1st and 2nd degree	332 (10.0)	178 (10.8)	82 (12.4)	72 (7.0)	
Missing	178 (5.4)	98 (6.0)	33 (5.0)	47 (4.6)	
Individual SES					
Education					
< High school	989 (29.7)	532 (32.4)	204 (30.9)	253 (24.7)	< 0.01
High school/certificate	1102 (33.1)	539 (32.8)	210 (31.8)	353 (34.5)	
≥ Diploma	1235 (37.1)	571 (34.8)	246 (37.3)	418 (40.8)	
Employment					
Unemployed/retired	1367 (41.1)	769 (46.8)	263 (39.9)	335 (32.7)	< 0.01
Employed	1941 (58.4)	865 (52.7)	396 (60.0)	680 (66.4)	
Missing	18 (0.5)	8 (0.5)	1 (0.2)	9 (0.9)	
Income					
< $52,000	1216 (36.6)	660 (40.2)	225 (34.1)	331 (32.3)	< 0.01
$52,000–$129,999	1271 (38.2)	569 (34.7)	275 (41.7)	427 (41.7)	
≥ $130,000	476 (14.3)	214 (13.0)	91 (13.8)	171 (16.7)	
Missing	363 (10.9)	199 (12.1)	69 (10.5)	95 (9.3)	
Number of cars					
None	132 (4.0)	68 (4.1)	20 (3.0)	44 (4.3)	< 0.01
1 car	1109 (33.3)	571 (34.8)	223 (33.8)	315 (30.8)	
2 cars	1407 (42.3)	691 (42.1)	271 (41.1)	445 (43.5)	
> 2 cars	601 (18.1)	255 (15.5)	142 (21.5)	204 (19.9)	
Missing	77 (2.3)	57 (3.47)	4 (0.6)	16 (1.6)	
Marital status					
Married^6^	2440 (73.4)	1217 (74.1)	500 (75.8)	723 (70.6)	0.04
Not married^7^	886 (26.6)	425 (25.9)	160 (24.2)	301 (29.4)	
Language speaks at home					
English	3149 (94.7)	1562 (95.1)	623 (94.4)	964 (94.1)	0.51
Other	177 (5.3)	80 (4.9)	37 (5.6)	60 (5.9)	
Private insurance					
Full insurance	2110 (63.4)	1097 (66.8)	423 (64.1)	590 (57.6)	< 0.01
No/part insurance	1215 (36.5)	544 (33.1)	237 (35.9)	434 (42.4)	
Missing	1 (0.0)	1 (0.1)	0 (0)	0 (0)	
Residential area factors					
Remoteness					
Major city	1969 (59.2)	967 (58.9)	399 (60.5)	603 (58.9)	0.88
Inner regional	804 (24.2)	393 (23.9)	159 (24.1)	252 (24.6)	
Outer regional/remote	553 (16.6)	282 (17.2)	102 (15.5)	169 (16.5)	
Accessibility to treatment					
High accessibility	2616 (78.7)	1273 (77.5)	525 (79.6)	818 (79.9)	0.29
Low accessibility	710 (21.4)	369 (22.5)	135 (20.5)	206 (20.1)	
Area disadvantages					
Least disadvantaged	737 (22.2)	384 (23.4)	147 (22.3)	206 (20.1)	0.38
Middle SES	2014 (60.6)	983 (59.9)	400 (60.6)	631 (61.6)	
Most disadvantaged	575 (17.3)	275 (16.8)	113 (17.1)	187 (18.3)	
Last negative screening facilities^8^ (*n* = 2145)					
Facility type					
Public	1625 (75.8)	1110 (74.1)	515 (79.6)	**–**	< 0.01
Private	518 (24.2)	388 (25.9)	130 (20.1)	**–**	
Missing	2 (0.1)	0 (0)	2 (0.3)	**–**	
Remoteness					
Major city	1367 (63.7)	947 (63.2)	420 (64.9)	**–**	0.53
Inner regional	458 (21.4)	319 (21.3)	139 (21.5)	**–**	
Outer regional/remote	320 (14.9)	232 (15.5)	88 (13.6)	**–**	
Area disadvantages					
Least disadvantaged	401 (18.7)	305 (20.4)	96 (14.8)	**–**	0.03
Middle SES	1248 (58.2)	845 (56.4)	403 (62.3)	**–**	
Most disadvantaged	427 (19.9)	299 (20.0)	128 (19.8)	**–**	
Missing	69 (3.2)	49 (3.3)	20 (3.1)	**–**	

^1^Screen-detected: patients detected by a positive screening, no matter if they had a negative screening previously

^2^Interval cancer: patients detected by symptoms, but the interval between the date of cancer diagnosis and the last negative screening was no more than 730 days (2 years)

^3^Other symptom-detected: patients detected by symptoms, and the interval between date of cancer diagnosis and the last negative screening (if there is any) was more than 730 days (2 years)

^4^*p* value: generated using Chi-square test between categories of breast cancer and each factor in the first column

^5^BMI: body mass index. Overweight defined as BMI ≥ 25

^6^Including those living as married

^7^Including those not living as married

^8^Only screen-detected and interval cancer patients who had a negative mammographic screening in Queensland before diagnosis (*n* = 2145) were included

### Area-level variables

Area-level variables included the patient’s residential area and the location of the facility where they received their last mammogram (Table 1), with both coded to the Statistical Area Level 2 (SA2), as defined by the 2011 Australian Statistical Geography Standard (ASGS) classification system [20].

For residential area factors, three variables were generated based on the SA2 code. First, SA2 areas were classified into three levels of remoteness (“Major city”, “Inner regional”, “Outer regional/remote”) based on access to services using the ASGS Remoteness Area measure [25]. Areas were also classified into two levels of accessibility to treatment (high: < 1 h, low: ≥ 1 h) based on the road travel time from the geographic centre of participants’ residential SA2 to the closest radiation facility [26]. The third residential variable was area disadvantage, which was measured using the 2011 Index of Relative Socioeconomic Disadvantage (IRSD), a census-based aggregate measure that summarises information about the economic and social conditions of people and households within a SA2 [27]. While the IRSD is typically reported by Quintiles, for this study, the quintiles were collapsed into three groups as ‘most disadvantaged (Quintile 1)’, ‘middle SES (Quintile 2–4)’, and ‘least disadvantaged (Quintile 5)’, to increase the numbers of observations in each category.

Information about the location of the last negative screening facility included remoteness and area disadvantage based on the SA2 code. In addition, facility type was categorised into public or private.

The individual and area-level variables included in this study were chosen to reflect factors related to disease biology, policy implications, or behavioural interventions.

### Statistical analysis

Three logistic regression models were constructed to estimate the odds of breast cancer being diagnosed as later stage (stage II–IV vs. stage I, Model 1), higher grade (grade 3 vs. grade 1–2, Model 2), or triple negative (Yes vs. No, Model 3) among screen-detected, interval, and other symptom-detected cancers. The models were adjusted for age at diagnosis, clinical breast and self-examination history, lifestyle, reproductive history, family history of breast and ovarian cancer, individual SES, and residential area factors (Table 1).

A fourth model considered only screen-detected and interval cancer patients who had a negative mammogram in Queensland before their subsequent breast cancer diagnosis. This model (Model 4) explored factors associated with being diagnosed with an interval cancer (rather than a subsequent screen-detected cancer) among this subgroup. Age at diagnosis, clinical breast and self-examination history, lifestyle, reproductive history, family history of breast and ovarian cancer, individual SES, residential area factors, and factors associated with the last negative screening facility were initially included in the model (Table 1).

Models 1–4 were further refined using a modified backward stepwise method [28]. All the available factors (listed in Table 1) were included in the models initially. A likelihood ratio test was used to drop variables with a conservative *p* value cutoff of > 0.2. At each step, variables previously removed from the model were tested to gauge their eligibility to be re-included into the model.

### Multiple imputation

Missing data ranged from 0 to 11.7% for different variables (Table 1). A complete case analysis would have excluded 25.3% of the initial cohort for Model 1–3 and 19.1% for Model 4, potentially introducing a bias if the excluded cases were a non-random sample. Missing data were handled with multiple imputation methods [29], using the Stata mi impute chained and mi estimate commands for chained equations and subsequent regression model estimation. In the imputation modelling, separately for each of the models 1–4, we included all the variables in the final model and the auxiliary variables that were correlated with missing variables (Pearson correlation > 0.4). Based on the percentage of incomplete cases [30], we performed 26 imputations for Models 1–3 and 20 imputations for Model 4.

### Sensitivity analysis

The classification of interval cancer and other symptom-detected cancer depends on the time interval between the date of last negative screening and date of diagnosis. Since only 20.3% of all symptom-detected patients noted they reported the exact date of their last negative screening (Appendix Table 1a), a sensitivity analysis was conducted to explore the potential impact of incorrect reporting on the study results.

The sensitivity analysis first identified those patients who may have been incorrectly classified into either interval or other symptom-detected categories. For example, if the calculated interval between a patient’s reported date of last negative screening and the date of diagnosis was 699 days (1 year 11 months), we classified this patient as “interval cancer”. However, if this patient indicated that her reported data were correct within plus or minus 6 months, her correct classification may have been “other symptom-detected”, since the real date of her previous screening could have been more than 2 years preceding the diagnosis. In the sensitivity analysis, we identified all such patients and randomly allocated half of them into the interval cancer group and the other half into the “other symptom-detected” group. Models 1–4 were re-run using the modified data to see if the observed patterns changed.

In addition, to evaluate the accuracy of recalled reproductive history, respondents were asked “How accurate is this age?” after questions like “At what age did you first get your periods?”; “Can you remember how old you were when you first went on a hormone contraceptive?”, with options of “exact age”, “within 1 year”, “within 2 years”, “within 5 years”, “Patients has no idea of their age”.

All statistical analyses were performed using Stata/SE version 16 (StataCorp, TX, USA).

## Results

A total of 3326 of 5426 eligible women were included in the main analysis (Model 1–3). The second analysis included only screen-detected and interval cancer patients who had a negative mammogram in Queensland before their subsequent breast cancer diagnosis (*n* = 2145, Model 4).

### Descriptive analysis

Among the 3,326 women in the main study cohort, 1,642 (49.4%) were classified as screen-detected, 660 (19.8%) were interval cancers, and 1,024 (30.8%) were other symptom-detected (Table 1). Overall, 50.1% were diagnosed with stage II–IV cancer, with this proportion being lowest among screen-detected patients (33.6%) and highest among other symptom-detected patients (68.6%). Similar patterns were observed according to tumour grade. There was a slight difference in patterns for women diagnosed with triple-negative cancers, with interval cancer patients having the highest proportion (13.0%) of triple-negative cancers and screen-detected cancers having the lowest (5.5%). Among other symptom-detected women who were invited for screening (aged 50–74 years), 22.3% had no history of breast screening.

### Severity at diagnosis

**Fig. 1:**
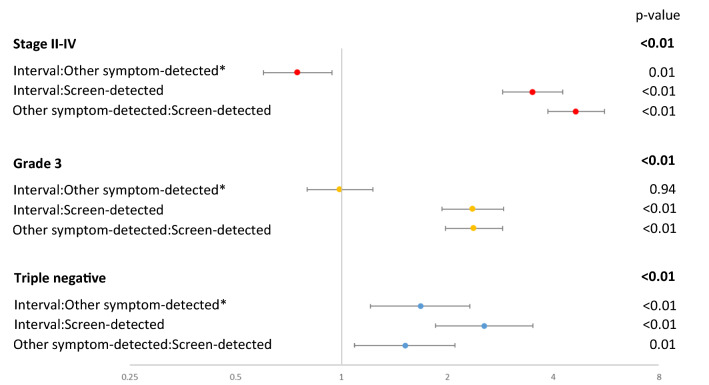
Odds ratios of breast cancer diagnosed with late stage, high grade, or triple negative by different methods of detection (screen-detected, interval cancer, and other symptom-detected). Results were adjusting for age at diagnosis, clinical breast and self-examination history, lifestyle, reproductive history, family history of breast and ovarian cancer, individual SES and residential area factors (Appendix Table 2); The overall *p* value for the independent variables in bold type is calculated using Wald tests to test the null hypothesis that all the coefficients of the independent variable are equal to zero; lines with asterisk were generated using the same model with different reference level

After adjusting for individual and area-level variables, compared with screen-detected breast cancer, women with interval cancers had higher odds of being diagnosed as stage II–IV (OR = 3.50, 2.9–4.3), high-grade (OR = 2.36, 1.9–2.9) and triple-negative (OR = 2.55, 1.9–3.5) breast cancers (Fig. 1, Appendix Table 2). Compared with other symptom-detected breast cancers, those diagnosed with interval cancers again had higher odds of having triple-negative (OR = 1.68, 1.2–2.3) cancers, but lower odds of being diagnosed with later stage breast cancers (OR = 0.75, 0.6–0.9) and there was no difference detected for grade (OR = 0.99, 0.8–1.2).

### Factors associated with interval cancer

**Fig. 2:**
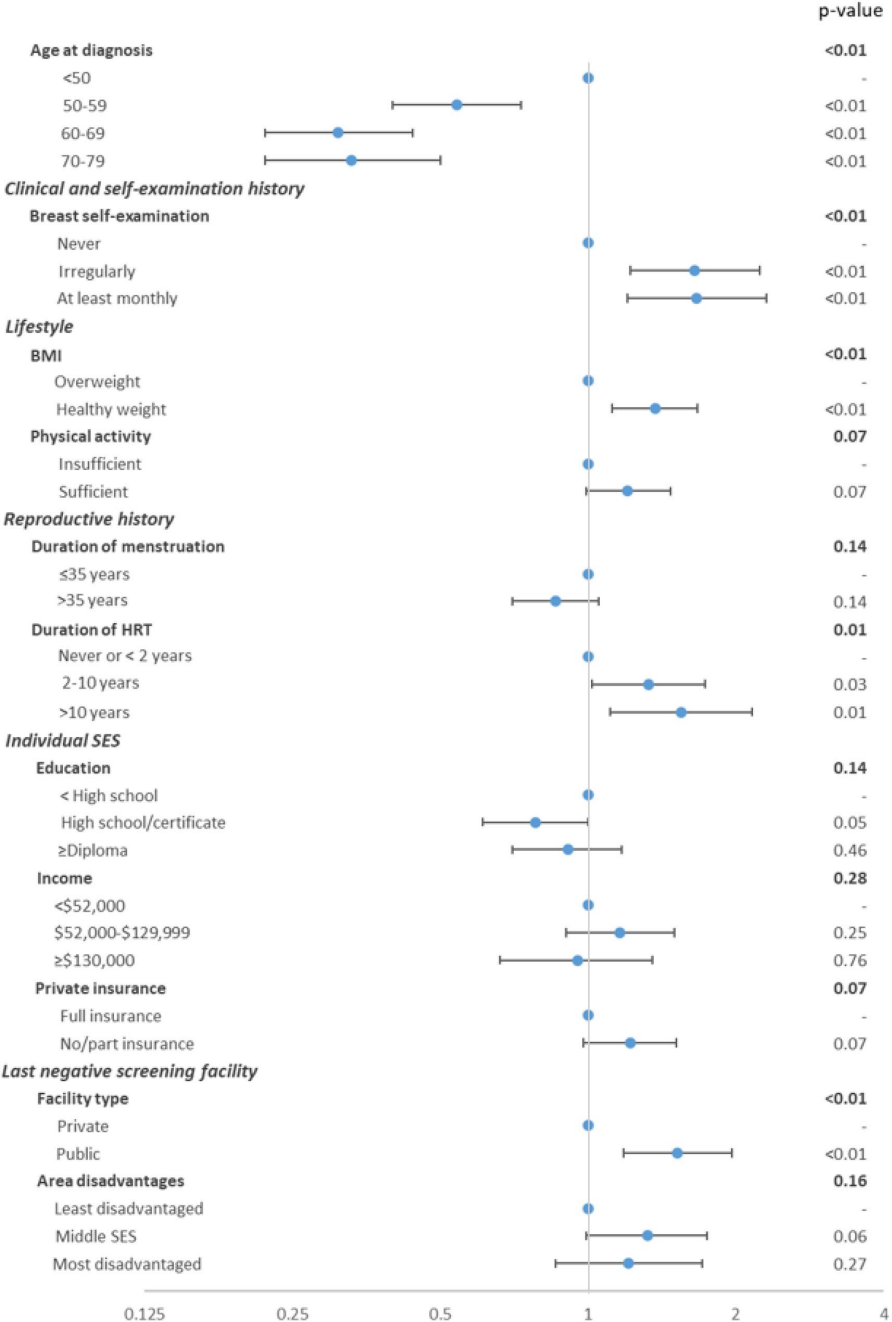
Odds ratios of interval breast cancer versus screen-detected cancer among women who had a negative mammogram prior to diagnosis (*n* = 2145). The overall *p* value for the independent variables in bold type is calculated using Wald tests to test the null hypothesis that all the coefficients of the independent variable are equal to zero

A total of 2,145 women reported having a negative mammogram prior to their breast cancer diagnosis. Of these, 69.8% were diagnosed at their next mammogram while 30.2% were diagnosed with an interval cancer. Overall, women had higher odds of being diagnosed with an interval cancer if they were younger age, practised breast self-examination (BSE), had a healthy weight before diagnosis, or had received HRT for more than 2 years prior to diagnosis (Fig. 2, Appendix Table 3). Women who had their previous negative mammogram in a public screening facility had higher odds of subsequently being diagnosed with an interval cancer compared to those whose negative mammogram was conducted in a private facility. There was no evidence that area disadvantage (*p* = 0.16) or remoteness (*p* > 0.20, removed from final model) of the facility location was associated with the risk of being diagnosed with an interval breast cancer.

### Sensitivity analysis

Given the uncertainty about recall of dates, among all the symptom-detected patients, 213 could have been allocated into either interval cancer or “other symptom-detected” categories. We randomly allocated 107 of them as interval cancer patients and the remaining 106 as “other symptom-detected”. The results from Models 1–4 for the revised dataset (Appendix Table 4 and 5) show similar patterns with Figs. 1 and 2.

From the response data about reproductive history, more than 90% of participants stated the accuracy for their menstruation and contraceptive use history was within 2 years; and more than 85% stated that the accuracy of their HRT histories was within 2 years (Appendix Table 1b).

## Discussion

This study found that, compared with screen-detected breast cancers, interval cancers were two or three times more likely to be advanced-stage, high-grade, and triple-negative subtypes. Of those breast cancers detected through symptoms, there was some evidence that women diagnosed with an interval breast cancer had a more favourable stage distribution than those diagnosed among women who had no history of screening in the previous 2 years (i.e. other symptom-detected breast cancers). Combined, these results highlight the benefit of screening in detecting breast cancers, including those diagnosed within 2 years of a negative screen.

To the best of our knowledge, this is the first study which has quantified the poorer severity of other symptom-detected cancers compared with interval cancers. In addition, our results are consistent with previous studies highlighting that, compared to screen-detected breast cancer, interval cancers are more likely to be higher grade, triple negative, and are associated with more advanced stage [3–9].

Consistent with previous studies, we found that younger patients [5, 17], women with a healthy weight prior to diagnosis [9] or had used HRT before diagnosis [9, 12–14] were more likely to be diagnosed with an interval cancer [5, 17]. Furthermore, this study found evidence of an ordinal trend for the duration effect of HRT, while most other studies have considered less detailed measures. Suggested reasons for an association with HRT use are that it may increase the growth of pre-existing cancers [31], or its association with increased breast density in some women [32] could result in reduced sensitivity of mammography and greater risk of false negative interval cancer. To our best knowledge, this study provides the first evidence that women who performed BSE at least monthly or who had their previous negative screening in a public facility were more likely to be diagnosed with interval cancer than those who had never conducted a BSE or women who had their previous screening in a private facility.

It is possible that women who performed BSE regularly were more likely to detect the tumour earlier than those who did not [33], as our data indicated that 67% of the interval cancer patients reported breast lump as their initial symptom. Our data also indicate that women with a family history of breast cancer were more likely to report conducting a regular BSE (results not shown).

At least in theory, BSE has the potential to be a cheap, non-complicated and non-invasive method for detecting breast cancer early [34]. However, there is ongoing debate regarding the efficacy of BSE in terms of mortality reduction [35, 36], and a number of organisations internationally, including in Australia, no longer recommend them as a screening method [34]. Although BSE currently is not an endorsed early detection behaviour according to Australian guidelines [37], our results suggest that further exploration of the utility of BSE might be warranted, particularly in relation to the detection of interval breast cancers.

Our study found that women who had their previous negative mammogram in a public screening facility had higher odds of subsequently being diagnosed with an interval cancer compared to those whose negative mammogram was conducted in a private facility. Unfortunately, there are no population-based data on the use of private screening facilities to assist with the interpretation of these results. However, that private mammograms involve substantial out-of-pocket expenses, along with the relatively high participation in free publicly funded mammograms, suggests that any private use would be small. Although data are lacking, a 2008 paper suggested about 20% of mammograms are carried out through private screening [38]). The lack of population-based data on mammogram screening rates in private facilities in Australia and information about the characteristics of women who attend private mammograms, limits the capacity to quantify all screening mammogram activities and assess their effectiveness. To address this gap, it is important to have processes in place to ensure these data are available, with appropriate safeguards for the confidentiality of the service providers and of the women accessing those services.

Between 2009 and 2013, the BreastScreen Queensland Program has undergone several developments. Consistent with evidence that digital mammography reduced the risk of interval cancer compared with screen-film mammography [14, 39–42], the reading methods began to change from film to digital from 2009 onward. In September 2012, BreastScreen Queensland introduced the Statewide Centrally Coordinated Reading Model, which allowed images acquired anywhere in the state to be read ‘anywhere in the state’ through electronic soft copy reading [personal communication, BreastScreen Queensland]. In particular, screen reading is only undertaken in one of eleven BreastScreen Queensland reading rooms or the Central Reading Hub in Brisbane. These standardised processes across the state likely explain at least part of the lack of geographical disparities in the detection of interval cancers in our study, since the odds of a women having an interval cancer were not associated with the area disadvantage and remoteness of the screening facilities.

Study strengths include the use of a population-based cohort of over three thousand women diagnosed with breast cancer, collecting information through questionnaires and access to medical records. Further, multiple imputation was used to enable all participants to be included in the final analysis, thus reducing the potential for additional biases by excluding records with missing data. Given the retrospective data collection and recognising the potential for recall bias in the screening dates, we were able to assess the potential impacts of reported accuracy by respondents in the sensitivity analyses.

This study has several limitations. First, the retrospective nature of this study increases the potential for inaccuracies in the information provided; however, the study participants had generally high confidence in their recalled reproductive history. In addition, we assessed the potential impacts of reported accuracy on the key results in the sensitivity analyses. Future studies using a prospective cohort design will minimise any recall bias and provide higher-level evidence for the current research questions. Second, to improve the performance of the logistic models, we combined stage II and stage III–IV to make the data more balanced. This leads to a heterogenous mix within the advanced stage (stage II–IV).

## Conclusion

These results highlight the benefits of screening to reduce the stage of breast cancers at diagnosis, even among interval cancers, and highlight the importance of reducing the nearly one in four women diagnosed with breast cancer who, although eligible, had no screening history in the 2 years prior to diagnosis. That women of healthy weight and who conduct monthly breast self-examinations were more likely to have an interval breast cancer than a screen-detected breast cancer may reflect their increased ability to notice lumps or other symptoms between screening intervals.

## Supplementary Information

Below is the link to the electronic supplementary material.

Supplementary file1 (DOCX 42 KB)
