# Supplementary material for: Severity and risk factors of interval breast cancer in Queensland, Australia: a population-based study
Source: Breast Cancer. 2023 Feb 21;30(3):466–77. doi: 10.1007/s12282-023-01439-4 (PMC10119209; doi:10.1007/s12282-023-01439-4)
Supplement: Supplementary file 1 — Supplementary file1 (DOCX 42 KB) [file 12282_2023_1439_MOESM1_ESM.docx]

Appendix Table 1a. Levels of accuracy of the last negative screening data among symptom-detected cancer patients (interval cancer and other symptom-detected cancer)

| **Accuracy** | **Interval cancer** | **Other symptom-detected** | **Total** |
| --- | --- | --- | --- |
| Exact date | 276 (41.8%) | 65 (6.4%) | 341 (20.3%) |
| Within one month | 320 (48.5%) | 146 (14.3%) | 466 (27.7%) |
| Within six months | 42 (6.4%) | 80 (7.8%) | 122 (7.2%) |
| Within one year | 14 (2.1%) | 125 (12.2%) | 139 (8.3%) |
| Within two years | 1 (0.2%) | 36 (3.5%) | 37 (2.2%) |
| “Best guess”^1^ | 7 (1.1%) | 107 (10.5%) | 114 (6.8%) |
| Missing^2^ | 0 (0.0%) | 465 (45.4%) | 465 (27.6%) |
| **Total** | **660 (100.0%)** | **1024 (100.0%)** | **1684 (100.0%)** |

*Note: 1. We assumed the accuracy of “best guess” was within three years. 2. Participants with missing values in levels of accuracy did not have a previous mammogram screen.*

Appendix Table 1b. Levels of accuracy of reproductive history

| Accuracy | Exact age | Within 1 year | Within 2 years | Within 5 years | No idea | Missing |
| --- | --- | --- | --- | --- | --- | --- |
| Age period started | 2104 (63.3%) | 1071 (32.2%) | 102 (3.1%) | 4 (0.1%) | 19 (0.6%) | 26 (0.8%) |
| Age period stopped^3^ | 1698 (60.6%) | 618 (22.1%) | 314 (11.2%) | 124 (4.4%) | 44 (1.6%) | 3 (0.1%) |
| Age started contraceptives^4^ | 1620 (55.6%) | 1002 (34.4%) | 210 (7.2%) | 42 (1.4%) | 32 (1.0%) | 7 (0.2%) |
| Age stopped contraceptives^4^ | 1584 (54.4%) | 796 (27.3%) | 327 (11.2%) | 116 (4.0%) | 87 (3.0%) | 3 (0.1%) |
| Age started HRT^5^ | 401 (36.5%) | 365 (33.2%) | 190 (17.3%) | 87 (7.9%) | 56 (5.1%) | 0 |
| Age stopped HRT^5^ | 553 (50.3%) | 279 (25.4%) | 131 (11.9%) | 48 (4.4%) | 68 (6.2%) | 20 (1.8%) |

*Note: 3.Among women whose period stopped (n=2801). 4. Among women who had used contraceptives (n=2913). 5. Among women who had used hormone replacement therapy (HRT) before breast cancer diagnosis.*

Appendix Table 2 Odds ratios of breast cancer diagnosed with late stage, high grade, or triple negative by different methods of detection.

|  | **Stage II-IV** | **Grade 3** | **Triple negative** |
| --- | --- | --- | --- |
| **Methods of detection** |  |  |  |
| Interval:Screen-detected | 3.50 (2.87-4.26)* | 2.36 (1.93-2.89)* | 2.55 (1.85-3.51)* |
| Interval:Other symptom-detected | 0.75 (0.60-0.94)* | 0.99 (0.80-1.23) | 1.68 (1.21-2.32)* |
| **Age at diagnosis** |  |  |  |
| <50 | 1 | 1 | 1 |
| 50-59 | 0.81 (0.64-1.01) | 0.82 (0.64-1.05) | 0.91 (0.62-1.35) |
| 60-69 | 0.57 (0.44-0.73)* | 0.71 (0.54-0.93)* | 0.94 (0.60-1.48) |
| 70-79 | 0.52 (0.38-0.71)* | 0.68 (0.49-0.93)* | 1.13 (0.67-1.90) |
| ***Clinical and self-examination history*** | | | |
| **Clinical breast examination** |  |  |  |
| Never | 1 | - | - |
| Irregularly | 0.79 (0.64-0.97)* | - | - |
| Yearly | 0.72 (0.56-0.91)* | - | - |
| **Breast self-examination** |  |  |  |
| Never | - | - | 1 |
| Irregularly | - | - | 0.73 (0.51-1.04) |
| At least monthly | - | - | 0.91 (0.62-1.34) |
| ***Lifestyle*** |  |  |  |
| **BMI^1^** |  |  |  |
| Healthy weight | 1 | 1 | - |
| Overweight | 1.46 (1.24-1.71)* | 1.14 (0.97-1.34) | - |
| **Smoking** |  |  |  |
| Never smoked | 1 | 1 | 1 |
| Used to smoke | 0.84 (0.72-0.99)* | 0.87 (0.73-1.02) | 0.77 (0.58-1.03) |
| Current smoker | 0.69 (0.52-0.93)* | 0.73 (0.55-0.98)* | 0.74 (0.45-1.21) |
| **Drinking** |  |  |  |
| <Once a month | - | 1 | - |
| ≥Once a month | - | 1.18 (1.00-1.39)* | - |
| ***Reproductive history*** |  |  |  |
| **Duration of menstruation** |  |  |  |
| ≤35 years | - | 1 | 1 |
| >35 years | - | 0.72 (0.61-0.86)* | 0.83 (0.62-1.12) |
| **Menopause** |  |  |  |
| No | 1 | 1 | 1 |
| Yes | 1.93 (1.53-2.42)* | 1.44 (1.14-1.81)* | 1.35 (0.92-1.97) |
| **Age started using contraceptives** |  |  |  |
| Never used | - | - | 1 |
| ≤18 | - | - | 1.78 (1.09-2.89)* |
| >18 | - | - | 0.98 (0.63-1.51) |
| **Duration of contraceptives using** |  |  |  |
| Never or <2 years | - | 1 | - |
| 2-10 years | - | 1.26 (1.01-1.57)* | - |
| >10 years | - | 1.30 (1.04-1.64)* | - |
| **Duration of HRT^2^** |  |  |  |
| Never or < 2 years | - | 1 | - |
| 2-10 years | - | 0.85 (0.67-1.08) | - |
| >10 years | - | 0.66 (0.48-0.90)* | - |
| ***Family history*** |  |  |  |
| Relatives with breast or ovarian cancer | |  |  |
| None | - | 1 | - |
| 2^nd^ degree | - | 1.04 (0.86-1.26) | - |
| 1^st^ degree | - | 0.66 (0.51-0.85)* | - |
| 1^st^ & 2^nd^ degree | - | 0.87 (0.67-1.13) | - |
| ***Individual SES*** |  |  |  |
| **Income** |  |  |  |
| <$52,000 | 1 | - | - |
| $52,000-$129,999 | 1.02 (0.84-1.24) | - | - |
| ≥$130,000 | 1.27 (0.97-1.66) | - | - |
| **Private insurance** |  |  |  |
| Full insurance | 1 | - | - |
| No/part insurance | 1.37 (1.16-1.61)* | - | - |
| ***Residential area factors*** |  |  |  |
| **Remoteness** |  |  |  |
| Major city | - | 1 | 1 |
| Inner regional | - | 1.25 (1.04-1.50)* | 1.19 (0.87-1.63) |
| Outer regional/remote | - | 1.12 (0.91-1.39) | 1.58 (1.15-2.19)* |

*1.BMI: body mass index. Overweight defined as BMI≥25; 2. HRT: hormone replacement therapy; *p<0.05.*

Appendix Table 3 Odds ratios of interval breast cancer versus screen-detected cancer among cancer patients who had a negative mammographic screening in Queensland before diagnosis (n=2145).

|  | **Odds ratio** | **95% CI** | **p-value^3^** |
| --- | --- | --- | --- |
| **Age at diagnosis** |  |  | **<0.01** |
| <50 | 1 | - | - |
| 50-59 | 0.54 | 0.40-0.73 | <0.01 |
| 60-69 | 0.31 | 0.22-0.44 | <0.01 |
| 70-79 | 0.33 | 0.22-0.50 | <0.01 |
| ***Clinical and self-examination history*** | | | |
| **Breast self-examination** |  |  | **<0.01** |
| Never | 1 | - | - |
| Irregularly | 1.65 | 1.22-2.24 | <0.01 |
| At least monthly | 1.66 | 1.20-2.31 | <0.01 |
| ***Lifestyle*** |  |  |  |
| **BMI^1^** |  |  | **<0.01** |
| Overweight | 1 | - | - |
| Healthy weight | 1.37 | 1.12-1.67 | <0.01 |
| **Physical activity** |  |  | **0.07** |
| Insufficient | 1 | - | - |
| Sufficient | 1.20 | 0.99-1.47 | 0.07 |
| ***Reproductive history*** |  |  |  |
| **Duration of menstruation** |  |  | **0.14** |
| ≤35 years | 1 | - | - |
| >35 years | 0.86 | 0.70-1.05 | 0.14 |
| **Duration of HRT^2^** |  |  | **0.01** |
| Never or < 2 years | 1 | - | - |
| 2-10 years | 1.33 | 1.02-1.73 | 0.03 |
| >10 years | 1.55 | 1.11-2.16 | 0.01 |
| ***Individual SES*** |  |  |  |
| **Education** |  |  | **0.14** |
| < High school | 1 | - | - |
| High school/certificate | 0.78 | 0.61-1.00 | 0.05 |
| ≥Diploma | 0.91 | 0.70-1.17 | 0.46 |
| **Income** |  |  | **0.28** |
| <$52,000 | 1 | - | - |
| $52,000-$129,999 | 1.16 | 0.90-1.50 | 0.25 |
| ≥$130,000 | 0.95 | 0.66-1.35 | 0.76 |
| **Private insurance** |  |  | **0.07** |
| Full insurance | 1 | - | - |
| No/part insurance | 1.22 | 0.98-1.51 | 0.07 |
| ***Last negative screening facility*** | |  |  |
| **Facility type** |  |  | **<0.01** |
| Private | 1 | - | - |
| Public | 1.52 | 1.18-1.96 | <0.01 |
| **Area disadvantages** |  |  | **0.16** |
| Least disadvantaged | 1 | - | - |
| Middle SES | 1.32 | 0.99-1.75 | 0.06 |
| Most disadvantaged | 1.21 | 0.86-1.71 | 0.27 |

*1.BMI: body mass index. Overweight defined as BMI≥25; 2.* *HRT: hormone replacement therapy; 3.* *The overall p-value for the independent variables in bold type is calculated using Wald tests to test the null hypothesis that all the coefficients of the independent variable are equal to zero.*

Appendix Table 4 Sensitivity analysis^1^ - Odds ratios of breast cancer diagnosed with late stage, high grade, or triple negative by different methods of detection.

|  | **Stage II-IV** | **Grade 3** | **Triple negative** |
| --- | --- | --- | --- |
| **Methods of detection** |  |  |  |
| Interval:Screen-detected | 3.41 (2.82-4.13)* | 2.24 (1.84-2.72)* | 2.41 (1.76-3.31)* |
| Interval:Other symptom-detected | 0.70 (0.56-0.88)* | 0.90 (0.73-1.11) | 1.55 (1.12-2.16)* |
| **Age at diagnosis** |  |  |  |
| <50 | 1 | 1 | 1 |
| 50-59 | 0.81 (0.65-1.02) | 0.83 (0.65-1.06) | 0.92 (0.62-1.37) |
| 60-69 | 0.57 (0.44-0.74)* | 0.71 (0.54-0.94)* | 0.93 (0.60-1.46) |
| 70-79 | 0.52 (0.38-0.72)* | 0.68 (0.49-0.93)* | 1.11 (0.65-1.89) |
| ***Clinical and self-examination history*** | | | |
| **Clinical breast examination** |  |  |  |
| Never | 1 | - | - |
| Irregularly | 0.80 (0.65-0.98)* | - | - |
| Yearly | 0.73 (0.57-0.92)* | - | - |
| **Breast self-examination** |  |  |  |
| Never | - | - | 1 |
| Irregularly | - | - | 0.72 (0.51-1.04) |
| At least monthly | - | - | 0.92 (0.62-1.35) |
| ***Lifestyle*** |  |  |  |
| **BMI^2^** |  |  |  |
| Healthy weight | 1 | 1 | - |
| Overweight | 1.46 (1.25-1.71)* | 1.14 (0.97-1.34) | - |
| **Smoking** |  |  |  |
| Never smoked | 1 | 1 | 1 |
| Used to smoke | 0.84 (0.72-0.99)* | 0.86 (0.73-1.02) | 0.76 (0.57-1.01) |
| Current smoker | 0.69 (0.52-0.92)* | 0.73 (0.54-0.97)* | 0.73 (0.45-1.20) |
| **Drinking** |  |  |  |
| <Once a month | - | 1 | - |
| ≥Once a month | - | 1.19 (1.01-1.39)* | - |
| ***Reproductive history*** |  |  |  |
| **Duration of menstruation** |  |  |  |
| ≤35 years | - | 1 | 1 |
| >35 years | - | 0.73 (0.61-0.87)* | 0.84 (0.63-1.11) |
| **Menopause** |  |  |  |
| No | 1 | 1 | 1 |
| Yes | 1.94 (1.54-2.45)* | 1.45 (1.15-1.82)* | 1.34 (0.92-1.96) |
| **Age started using contraceptives** |  |  |  |
| Never used | - | - | 1 |
| ≤18 | - | - | 1.79 (1.11-2.90)* |
| >18 | - | - | 0.98 (0.64-1.52) |
| **Duration of contraceptives using** |  |  |  |
| Never or <2 years | - | 1 | - |
| 2-10 years | - | 1.26 (1.01-1.56)* | - |
| >10 years | - | 1.30 (1.04-1.63)* | - |
| **Duration of HRT^3^** |  |  |  |
| Never or < 2 years | - | 1 | - |
| 2-10 years | - | 0.85 (0.67-1.08) | - |
| >10 years | - | 0.67 (0.49-0.93)* | - |
| ***Family history*** |  |  |  |
| Relatives with breast or ovarian cancer | |  |  |
| None | - | 1 | - |
| 2^nd^ degree | - | 1.05 (0.87-1.28) | - |
| 1^st^ degree | - | 0.66 (0.51-0.85)* | - |
| 1^st^ & 2^nd^ degree | - | 0.87 (0.67-1.13) | - |
| ***Individual SES*** |  |  |  |
| **Income** |  |  |  |
| <$52,000 | 1 | - | - |
| $52,000-$129,999 | 1.02 (0.84-1.24) | - | - |
| ≥$130,000 | 1.25 (0.96-1.62) | - | - |
| **Private insurance** |  |  |  |
| Full insurance | 1 | - | - |
| No/part insurance | 1.36 (1.15-1.60)* | - | - |
| ***Residential area factors*** |  |  |  |
| **Remoteness** |  |  |  |
| Major city | - | 1 | 1 |
| Inner regional | - | 1.25 (1.04-1.51)* | 1.19 (0.87-1.62) |
| Outer regional/remote | - | 1.12 (0.91-1.39) | 1.55 (1.12-2.14)* |

*1.* *Screen-detected patients are the same with the original data analysis classification; Patients with a chance of misclassification (n=213) were randomly allocated to interval cancer (n=107) and other symptom-detected cancer (n=106). 2. BMI: body mass index. Overweight defined as BMI≥25; 3. HRT: hormone replacement therapy; *p<0.05.*

Appendix Table 5 Sensitivity analysis^1^ – Odds ratios of interval breast cancer versus screen-detected cancer among cancer patients who had a negative mammographic screening in Queensland before diagnosis (n=2145).

|  | **Odds ratio** | **95% CI** | **p-value^4^** |
| --- | --- | --- | --- |
| **Age at diagnosis** |  |  | **<0.01** |
| <50 | 1 | - | - |
| 50-59 | 0.54 | 0.40-0.73 | <0.01 |
| 60-69 | 0.31 | 0.22-0.43 | <0.01 |
| 70-79 | 0.32 | 0.21-0.49 | <0.01 |
| ***Clinical and self-examination history*** | | | |
| **Breast self-examination** |  |  | **<0.01** |
| Never | 1 | - | - |
| Irregularly | 1.65 | 1.22-2.24 | <0.01 |
| At least monthly | 1.66 | 1.20-2.30 | <0.01 |
| ***Lifestyle*** |  |  |  |
| **BMI^2^** |  |  | **<0.01** |
| Overweight | 1 | - | - |
| Healthy weight | 1.37 | 1.12-1.68 | <0.01 |
| **Physical activity** |  |  | **0.06** |
| Insufficient | 1 | - | - |
| Sufficient | 1.21 | 0.99-1.47 | 0.06 |
| ***Reproductive history*** |  |  |  |
| **Duration of menstruation** |  |  | **0.14** |
| ≤35 years | 1 | - | - |
| >35 years | 0.85 | 0.69-1.05 | 0.14 |
| **Duration of HRT^3^** |  |  | **0.01** |
| Never or < 2 years | 1 | - | - |
| 2-10 years | 1.34 | 1.03-1.74 | 0.03 |
| >10 years | 1.56 | 1.12-2.18 | 0.01 |
| ***Individual SES*** |  |  |  |
| **Education** |  |  | **0.14** |
| < High school | 1 | - | - |
| High school/certificate | 0.78 | 0.61-1.00 | 0.05 |
| ≥Diploma | 0.91 | 0.71-1.18 | 0.48 |
| **Income** |  |  | **0.23** |
| <$52,000 | 1 | - | - |
| $52,000-$129,999 | 1.16 | 0.91-1.49 | 0.24 |
| ≥$130,000 | 0.92 | 0.65-1.32 | 0.66 |
| **Private insurance** |  |  | **0.08** |
| Full insurance | 1 | - | - |
| No/part insurance | 1.21 | 0.98-1.50 | 0.08 |
| ***Last negative screening facility*** | |  |  |
| **Facility type** |  |  | **<0.01** |
| Private | 1 | - | - |
| Public | 1.51 | 1.17-1.95 | <0.01 |
| **Area disadvantages** |  |  | **0.16** |
| Least disadvantaged | 1 | - | - |
| Middle SES | 1.32 | 0.99-1.77 | 0.06 |
| Most disadvantaged | 1.23 | 0.87-1.75 | 0.24 |

*1. 1.* *Screen-detected patients are the same with the original data analysis classification; Patients with a chance of misclassification (n=213) were randomly allocated to interval cancer (n=107) and other symptom-detected cancer (n=106). 2.* *BMI: body mass index. Overweight defined as BMI≥25; 3.* *HRT: hormone replacement therapy; 4. The overall p-value for the independent variables in bold type is calculated using Wald tests to test the null hypothesis that all the coefficients of the independent variable are equal to zero.*
